# How facial expressions reveal acute pain in domestic animals with facial pain scales as a diagnostic tool

**DOI:** 10.3389/fvets.2025.1546719

**Published:** 2025-03-04

**Authors:** Daniel Mota-Rojas, Alexandra L. Whittaker, Genaro A. Coria-Avila, Julio Martínez-Burnes, Patricia Mora-Medina, Adriana Domínguez-Oliva, Ismael Hernández-Avalos, Adriana Olmos-Hernández, Antonio Verduzco-Mendoza, Alejandro Casas-Alvarado, Temple Grandin

**Affiliations:** ^1^Neurophysiology, Behavior and Animal Welfare Assessment, DPAA, Universidad Autónoma Metropolitana (UAM), Mexico City, Mexico; ^2^School of Animal and Veterinary Sciences, Roseworthy Campus, University of Adelaide, Roseworthy, SA, Australia; ^3^Instituto de Investigaciones Cerebrales, Universidad Veracruzana, Xalapa, Mexico; ^4^Instituto de Ecología Aplicada, Facultad de Medicina Veterinaria y Zootecnia, Universidad Autónoma de Tamaulipas, Victoria City, Mexico; ^5^Facultad de Estudios Superiores Cuautitlán, Universidad Nacional Autónoma de México (UNAM), Cuautitlán, Mexico; ^6^Division of Biotechnology-Bioterio and Experimental Surgery, Instituto Nacional de Rehabilitación Luis Guillermo Ibarra Ibarra (INR-LGII), Mexico City, Mexico; ^7^Department of Animal Science, Colorado State University, Fort Collins, CO, United States

**Keywords:** pain, facial expressions, neurobiology, acute pain, grimace

## Abstract

The growing interest in managing and recognizing pain in animals has led to the search for more sensitive methods to evaluate it, especially because some species conceal any visible changes associated with pain or are not easily assessed. Research has shown that an animal's facial expression changes when exposed to painful stimuli. Thus, developing several pain scales (grimace scales) in species such as horses, cattle, pigs, sheep, donkeys, rabbits, rats, mice, and cats has helped to improve the study of pain in veterinary medicine. The possibility of using facial expression as an indicator of pain is due to the direct relationship between the activation of different regions of the Central Nervous System such as the somatosensory cortex, prefrontal cortex, amygdala, hippocampus, and hypothalamus, and their connections with the motor cortex to elicit motor responses including the movement of facial muscles. The present review aims to discuss the neurobiological association between acute pain and facial expressions in animals. It will analyze the importance of facial expression characterization and the use of grimace scales in farm, companion, and laboratory species.

## 1 Introduction

The study of pain in animals is a field of interest for animal welfare due to the ethical and legal obligations to minimize animal suffering (1–3), and prevent the chronification of pain (4, 5). It is known that animals perceiving pain show behavioral, emotional, or physiological changes that can be assessed through ethograms, blood sampling, and evaluation of the posture or movement (6). Moreover, changes in body language, including facial expression, have also been reported in animal species such as farm, companion, and laboratory animals (7–10). Facial expressions related to pain are considered a non-invasive method to recognize and manage pain even in those species considered stoic, who tend to conceal behavioral changes to avoid predation (11). Thus, they are part of the non-verbal repertoire of animals to communicate their state (12).

Facial expressions comprise a series of changes in the face, modulated by the contraction or relaxation of facial muscles (e.g., frowning in humans) (13). Facial expressions play a fundamental role during social interaction (14, 15), which is why they are considered indicators of the emotional state in both humans and non-human animals (12, 16, 17). However, the main clinical application that has been found in veterinary medicine is the development of several scales that use changes in facial expression to assess pain: the grimace scales. To date, grimace scales have been developed for horses (18, 19), cattle (20), pigs (21), sheep (22), rats (23), mice (24), rabbits (25), and cats (26).

Grimace scales use facial action units (FAU) or specific muscular movements that change the position of the ears and whiskers, tighten the eyes, and open the nostrils, among other changes when perceiving pain. After a noxious stimulus is recognized by peripheral and central pathways, its integration and efferent response require the participation of the motor cortex (27, 28). The motor cortex modulates the changes in facial expression due to its connection with the facial nerve, a structure that innervates all the muscles that modify facial expression (also called mimetic muscles) (29, 30). The present paper aims to review the neurobiological association between acute pain and facial expressions in animals. It will analyze the importance of facial expression characterization and the use of grimace scales in farm, companion, and laboratory animals.

## 2 The study of pain in animals through facial expression

The first mention of facial expressions in animals was made in Darwin's thesis (31), where he mentioned that, similarly to humans, non-human animals change their facial expressions according to their affective state (11, 32). Although emotional recognition is challenging in animals—because they cannot verbally express their feelings or mental state—(33–35), changes in the facial expression of animals have been used to assess the emotional dimension of pain (11, 26, 36–39).

**Figure 1:**
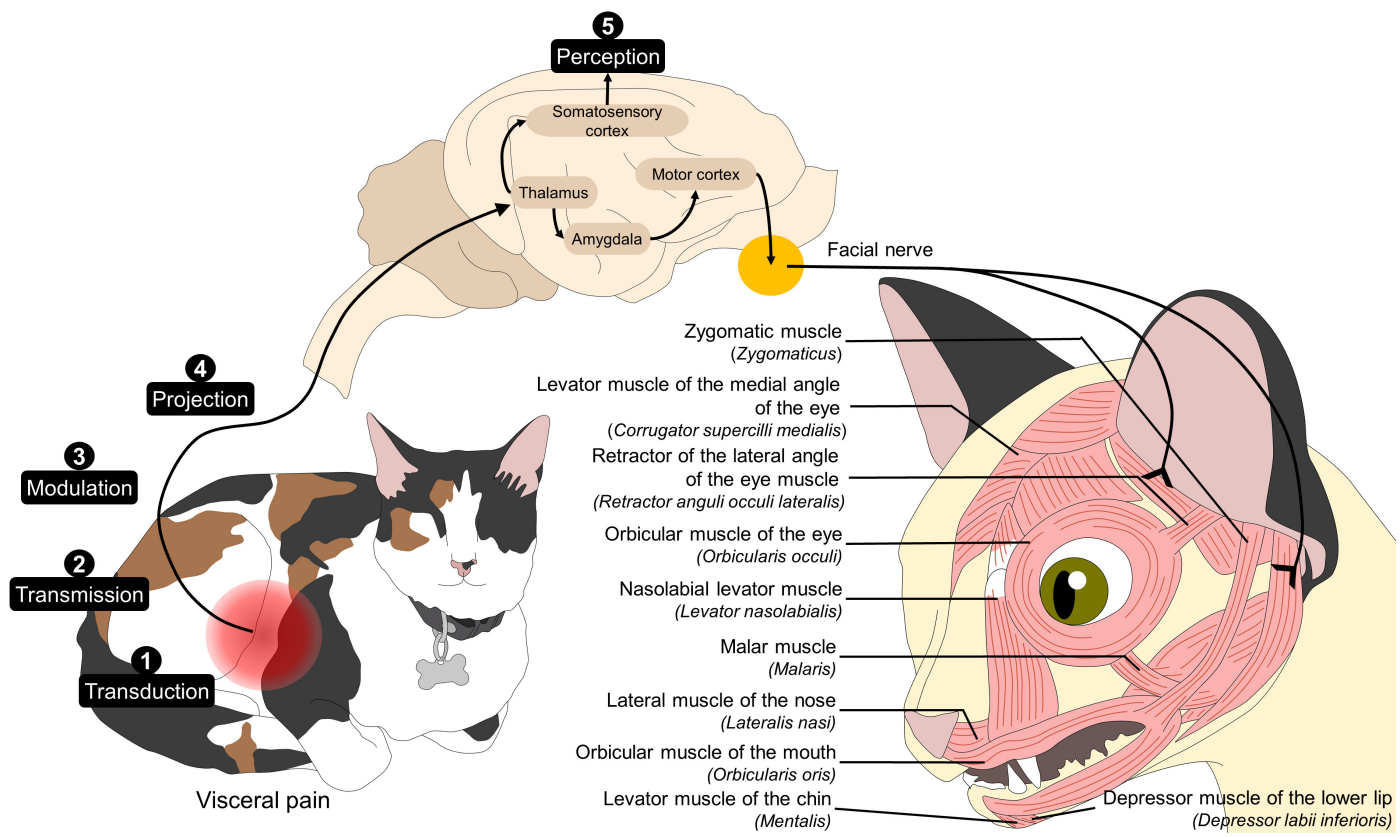
Association between pain neurobiology and facial expression. Noxious stimuli are transduced through free nerve endings. The nociceptive signals are transmitted by peripheral nerve fibers and modulated and projected from the dorsal horn of the spinal cord to various regions of the central nervous system. After reaching cerebral structures such as the thalamus, its connections to the amygdala and, consequently, to the motor cortex star the efferent motor response to pain. The motor cortex directly connects to the facial nucleus in the pons and, in turn, to the cranial nerve VII, which innervates the facial muscles. Activating these regions leads to the production of facial muscle movement that generate facial expressions that can be associated with the perception of pain.

To understand the association between facial expression and pain it is necessary to understand the nociceptive pathway and the connection to the cerebral centers that control facial muscles, as schematized in Figure 1. Pain perception requires the transduction, transmission, modulation, and projection of noxious stimuli from peripheral nerve endings to the central nervous system (40–45). In particular, the amygdala modulates the emotional dimension of pain and projects adrenergic fibers to the primary motor cortex, ventrolateral motor cortex, and the supplementary motor area (29, 46, 47). These areas innervate and coordinate the movement of facial muscles through the trigeminal and facial nerves (V and VII cranial nerves, respectively) (28). The activation of the facial nerve regulates the contraction of facial muscles, which modulates facial expressions. Meanwhile, the trigeminal nerve contributes to this process through jaw movements, such as jaw opening, and modifying facial expression in response to the animal's emotional state during the perception of acute pain (48).

Dolensek et al. (49) found that facial expression is a form of non-verbal communication that can convey features such as the intensity, valence, and persistence of certain emotions, including pain (2, 16, 50). Moreover, Bloom et al. (51, 52) reported that, by looking at the facial expressions of dogs, undergraduate students can differentiate and classify between sadness, happiness, anger, fear, surprise, and disgust (38, 53). Current research regarding pain in animals has shown that, for example, in horses, an equine pain face is characterized by “low” and/or “asymmetric” ears, an angled appearance of the eyes, dilated nostrils and tension of the muscles around the lips and chin. It is also characterized by a tense stare, described as tightened muscles around the eye, giving an angled appearance of the upper eyelid; additionally, the sclera at the medial canthus of the eyes is exposed and the animal stares intensively at an object or person when perceiving pain (54). Similarly, in cats, Holden et al. (55) reported that a feline facial expression is recognized with an accuracy of 98% by the ear position and movement around the muzzle.

The adaptation of the Facial Coding System (FACS), initially developed for humans, has helped in veterinary medicine to develop the grimace scales (although FACS and grimace scales have different purposes and FACS only evaluates facial movements without attributing them to a certain state) (56–58). Grimace scales consider anatomical differences according to the species. Some researchers prefer to use the term facial pain scales instead of grimace scales. For example, cats and mice in pain show whisker changes due to the contraction of the nasolabial levator muscle (*levator nasolabialis)* (48, 59). In contrast, this FAU is not reported in equines and bovines, where the tension of facial muscles around the muzzle (caused by the contraction of the *platysma* muscle) is more relevant (20, 54).

**Figure 2:**
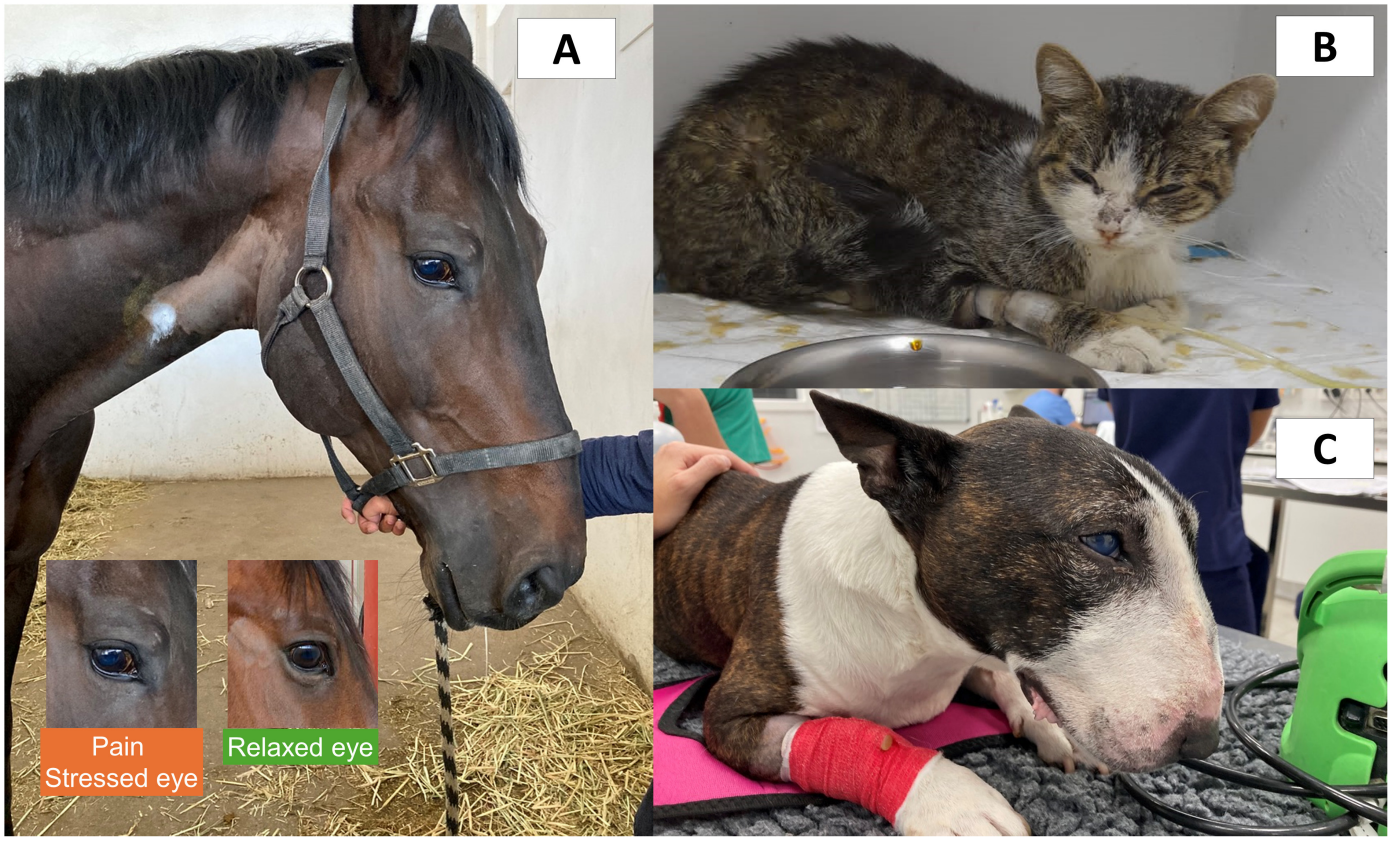
Comparison of facial expression associated with pain in different species. **(A)** A male quarter horse with traumatic laminitis. Changes in facial expression such as tension of the upper orbital muscles, tension of the lower lip, dilation of the nostrils, and tension of the facial muscles can be observed. The lower rectangles emphasize the changes observed in a horse with pain. When frightened, anxious, and in pain, the extraorbital eyelid muscles tend to hold more tension and create a “beak” over the eye. In contrast, the upper eyelid of the eye pulls up in a triangular shape due to muscle tension of the levator muscle of the medial angle of the eye (*m. levator anguli oculi medialis*). **(B)** Five-year-old male cat with acute pain due to interstitial cystitis. Changes in facial expression can be seen such as tension of the orbital muscles, head with a low position, tension in the whisker muscles, and slight flattening of the ears to the side. **(C)** Six-year-old Bull Terrier dog with acute pain due to pericardial effusion. In this picture, the tension of the orbital muscles, low head position, the tension of the facial muscles, retraction of the corner of the lip, and flattening of the ears can be observed. Images provided by the Equine Hospital of the Faculty of Higher Studies Cuautitlán, and to the Specialty Hospital of the Catholic University of Valencia.

Likewise, although in most species orbital region opening, caused by the contraction of the orbicular muscle of the eye (*orbicularis oculi*), is described, this is different according to the species (11, 18, 20, 60). Moreover, several scientific publications have made the error of confusing orbital tightening with orbital region opening. Figure 2 shows a comparison of images taken by the present authors focusing on the facial expressions associated with painful conditions in horses, dogs, and cats. It can be observed that the facial characteristics of pain differ according to the species. Consequently, to recognize the facial expressions associated with pain, it is necessary to study it separately by species and differentiate it from some other negative states such as fear and anxiety.

## 3 Grimace scales and facial expressions related to pain in farm animals

### 3.1 Horses

The Horse Grimace Scale (HGS) for domestic horses (*Equus caballus*) was developed by Dalla Costa et al. (18) to evaluate pain in animals undergoing castration. The HGS uses six FAU to characterize the pain expression of horses, including (1) stiffly backwards ears; (2) orbital region opening; (3) tension above the eye area; (4) prominent strained chewing muscles; (5) mouth strained and pronounced chin; and (6) strained nostrils and flattening of the profile (18). This scale rates pain from 0 to 2, where 0 means that facial expression changes are not present, while a score of 2 represents obviously present facial changes, with the highest possible score of 12 points. In Dalla Costa et al. (18) study, animals undergoing castration had significantly greater HGS scores (between 5 and 6 points) than animals receiving indolent procedures, regardless of the analgesic protocol.

One of the first reports was made by Flores et al. (61) in an experimental model of nociceptive mechanical stimulation in Friesians, Quarter Horses, and Warmbloods. After the authors induced mechanical nociception by stepped pressure at the level of the metacarpus, significant increases in the HGS scores (up to 7 points) were recorded, along with a decrease in parasympathetic tone activity assessed via heart rate variability. Similarly, Gleerup et al. (54) used another mechanical noxious stimulus—pressure from a tourniquet on the forearm—and a chemical nociceptive stimulus through the topical application of capsaicin to assess pain. It was reported that asymmetrical ears, the angled appearance of the eyes, tense stare, mediolaterally dilated nostrils, and tensed lips were present in horses with pain, who also had a mean pain score of up to 10 points during the application of the tourniquet. In other painful conditions such as laminitis, photographs and videos of horses with this condition were used to obtain the HGS scores. The scores showed significant differences before and after receiving treatment, decreasing from a basal score of 5.8 ± 2.0 to 3.5 ± 2.3 points (19).

During surgical procedures such as castration, the application of the HGS showed that animals having a mean score of 2 points were those that received a single analgesic drug while those that received multimodal analgesia recorded ~1.5 points at 20 h after castration (62). In dental disorders, Marcantonio Coneglian et al. (63) applied the HGS to identify acute pain. Using photographs of the animals to rate the pain into no pain, mild, moderate, and severe pain (0–3 points), it was reported that the pain score of the HGS decreased after dental treatment, but that training and face-to-face evaluation are required to accurately assess the pain level. The importance of standardized training to improve HGS accuracy was discussed by Dai et al. (64), who concluded that 30-min training is not enough to improve the recognition of facial changes in horses. Another example of the importance of integrating the HGS with other scales is Ask et al. (65) study, where an equine model of orthopedic pain was assessed using the HGS, the Equine Utrecht University Scale of Facial Assessment of Pain, the Equine Pain Scale, and the Composite Orthopedic Pain Scale. Although the HGS identified increases of up to 7 points after pain induction, the composite scale had the highest reliability (0.753) while the HGS had a coefficient of 0.522. Additionally, Ferlini Agne et al. (66) reported that the HGS does not differentiate between horses with and without gastric lesions, with a mean pain score of 2.76–3.95 and 1.79–4.20, respectively.

The HGS has also been used to evaluate other conditions that are not associated with pain but represent a negative state, such as distress. In this sense, the HGS was used to evaluate the level of discomfort in riding school horses. When comparing “rest” values with those obtained during “hard work,” significant increases in HGS score were obtained (from 3.30 ± 1.28 to 4.88 ± 1.47 points). However, the appearance of FAU such as backward ears and tension or stiffness in the muscles around the eyes might be present due to tiredness instead of pain (67). Similarly, Paladino et al. (68) used the HGS to evaluate the stress response in horses during assisted activities. No statistical differences were recorded before and after the assisted sessions, suggesting that these activities do not have adverse effects when using facial expressions to assess the level of distress. In contrast, another study reported that the HGS can identify “fear faces” when using the HGS in riding horses (69). However, as mentioned by Ijichi et al. (67), the anatomical structure and physiology must be considered when applying the HGS in other contexts that are not specifically related to pain.

Apart from the HGS, other pain scales that also consider facial traits have been developed in horses. The Equine Utrecht University Scale for Facial Assessment of Pain (EQUUS-FAP) was developed by VanDierendonck and van Loon (70), to assess visceral pain. This scale considers nine evaluation traits: (1) head movement; (2) eyelids; (3) focus; (4) nostrils; (5) corners of the mouth or lips; (6) muscle tone head; (7) flehming; (8) teeth grinding; and (9) ear position. With the EQUUS-FAP, it was identified that equines with visceral pain due to acute colic had a sensitivity and specificity of 87.5 and 88.0% to recognize pain (70). Similarly, in animals with orthopedic trauma or injury, the EQUUS-FAP recorded high pain scores (up to 6 points) that decreased with analgesic treatment (~4 points) (71).

The extensive nature of pain recognition through facial expressions in horses has allowed us to establish a solid base of studies that validate its effectiveness in different conditions –even in those related to distress instead of pain (72)–, in addition to adaptations to other equine species such as the donkey (*Equus asinus)* (73).

### 3.2 Piglets and sows

The Piglet Grimace Scale was developed by Viscardi et al. (74) to evaluate painful conditions such as castration and tail docking in domestic piglets (*Sus scrofa domestica*). The authors described three FAUs to characterize a facial expression of pain: (1) ear position; (2) cheek tightening/nose bulge; and (3) orbital region opening. Nonetheless, further studies considered 7 additional FAUs; (4) temporal tension; (5) tension above the eyes; (6) upper lip contraction: (7) snout angle; (8) forehead profile; (9) lower jaw profile; (9) snout place changes; and (10) nostril dilation (75). The FAUs are scored on a scale from 0 to 2 according to the presence of the facial changes (74). This scale arose to address routinely performed procedures known to be painful to piglets at a young age, including caudectomy, castration, dental resection, and ear mutilations or branding (11, 21, 74–76).

Di Giminiani et al. (75) used the PGS to assess pain in piglets undergoing tail docking and castrations. It was found that orbital region opening was the only FAU that significantly changed before tail docking while no differences were found during castration. Using the same routine procedures, Viscardi et al. (74) reported that high PGS scores were observed in piglets after castration and tail docking (up to 0.6 points) and that the scores did not decrease with the administration of topical anesthetics or injectable analgesics. These scores were also related to behavioral changes associated with pain such as tail wagging and isolation (74). In an attempt to refine tail docking with the use of the CO_2_ surgical laser technique, Lou et al. (77) found that this technique decreases the PGS scores (1.9 ± 0.1) when compared to tail docking with side pliers (2.3 ± 0.1). These results were also correlated with less tissue damage and reduced inflammation, indicating that the PGS can serve to recognize pain and identify less painful procedures for farm animals.

In the case of adult pigs, to date, there is not a validated grimace scale specifically designed for growing pigs. However, using the PGS as a base, Vullo et al. (21) determined that pigs undergoing surgical interventions due to cryptorchidism had higher mean PGS scores (2.16 ± 0.89) than before the procedure (1.02 ± 0.90), with an excellent inter-observer reliability (coefficient of 0.87). Moreover, Navarro et al. (78) developed a facial expression scale identifying five FAUs: (1) tension above the eyes; (2) snout angle; (3) neck tension; (4) temporal tension and ear position; and (5) cheek tension. By scoring the pain from 0 to 2, the authors reported high reliability of all proposed FAUs during farrowing with Kappa coefficients ranging from 0.63 to 0.90. Although additional studies evaluating adult pigs are required, adopting the PGS and considering specific FAUs for sows and pigs might also be helpful to evaluate other negative events such as thermal stress, as reported by Nie et al. (79). In this study the authors found that the mean average precision to identify facial expressions related to heat stress reached 92.3%, which might provide an additional tool to promote welfare during routine procedures in pigs.

### 3.3 Sheep and lambs

The Adult Sheep Grimace Scale (SGS) considers three FAUs: (1) orbital region opening; (2) ears and head position; and (3) flehming. Häger et al. (80), the developers of this scale, used SGS to assess postoperative pain after unilateral tibia osteotomy. SGS scores increased at 6 h after the surgery from 0.6 ± 0.2 to 1.9 ± 0.2 points and persisted with this intensity until day 17. The face of severe pain was characterized by severe orbital region opening, ear flattening, flehming, and hanging head. Particularly, the flehming reflex was associated with an intense sensation of pain.

Additionally, in ewes with mastitis, the SGS was used by Hussein and Al-Naqsshabendy (81), together with readings of the surface temperature of the eyes, ears, and nose, and cortisol values. Associations with higher SGS mean scores (up to 6), decreases in temperature (up to 9.6°C), and increases in cortisol (44.17 ± 7.9 nmol/l) were found in infected animals, concluding that facial expression is a useful tool to evaluate pain in sheep. Similarly, in an experimental orthopedic model in sheep, the SGS was used to determine the severity of the surgical intervention. After tendon ablation in German black-headed mutton ewes, SGS significantly increased to 1.3 ± 0.4 points (basal values of 0.8 ± 0.5) immediately after the procedure and remained high on the following days (up to 1.2 ± 0.5). These scores were accompanied by a relative increase of 23% in heart rate (82), which suggest that both facial and physiological changes are present in animals perceiving pain.

Another attempt to evaluate pain in sheep using changes in facial expression was made by McLennan and Mahmoud (83), the Sheep Pain Facial Expression Scale (SPFES). This scale comprises six FAUs, including (1) orbital region opening; (2) cheek tightening; (3) ear frontal position; (4) ear side position; (5) lip and jaw profile; and (6) nostril and philtrum shape. Using the SPFES, authors recorded higher scores in animals with painful diseases such as footrot and mastitis. In the case of animals with footrot, significantly higher scores were obtained before antibiotic treatment (4 points), in comparison with 90 days after treatment (2 points). Similarly, in ewes with mastitis, the highest scores were recorded before treatment with systemic antibiotics (4 *vs*. 2 at day 42). Yiting et al. (84) also used the SPFES and established a technique to detect FAUs including the ear position (AU1), the shape of the nose (AU4), and the opening of the eyes (AU7). By characterizing an ovine pain face as an individual with ears flipped and pinna not visible, with a “V” shaped nose, and eyes partly closed, this automated technique was able to estimate pain with an accuracy of 67%, showing promising alternatives to assess pain in sheep. It has been mentioned that the SPFES has an accuracy of up to 84% and an overall interrater reliability of 0.86, which makes this scale a reliable method to assess pain in sheep (83).

In the case of lambs, several routine procedures might cause pain due to the lack of analgesic administrations in these species. The adaptation of a pain scale in lambs has focused on its application in animals undergoing caudectomy. Guesgen et al. (85) developed the Lamb Grimace Scale (LGS) through a rubber ring caudectomy model in 5–6 week-old lambs. The LGS considers five FAUs: (1) orbital region opening; (2) nose features; (3) mouth features; (4) cheek flattening; and (5) ear posture, scoring each FAU on a three-point scale (0 to 2). The authors found that orbital region opening and mouth features greatly changed after tail docking, with an overall increase of LGS of 1.06 ± 0.11 (basal values of 0.34 ± 0.11). However, limited studies have been published using the LGS and are required to establish the usefulness of the scale in other painful conditions such as orthopedic issues or castration.

### 3.4 Cattle, bulls, and calves

**Figure 3:**
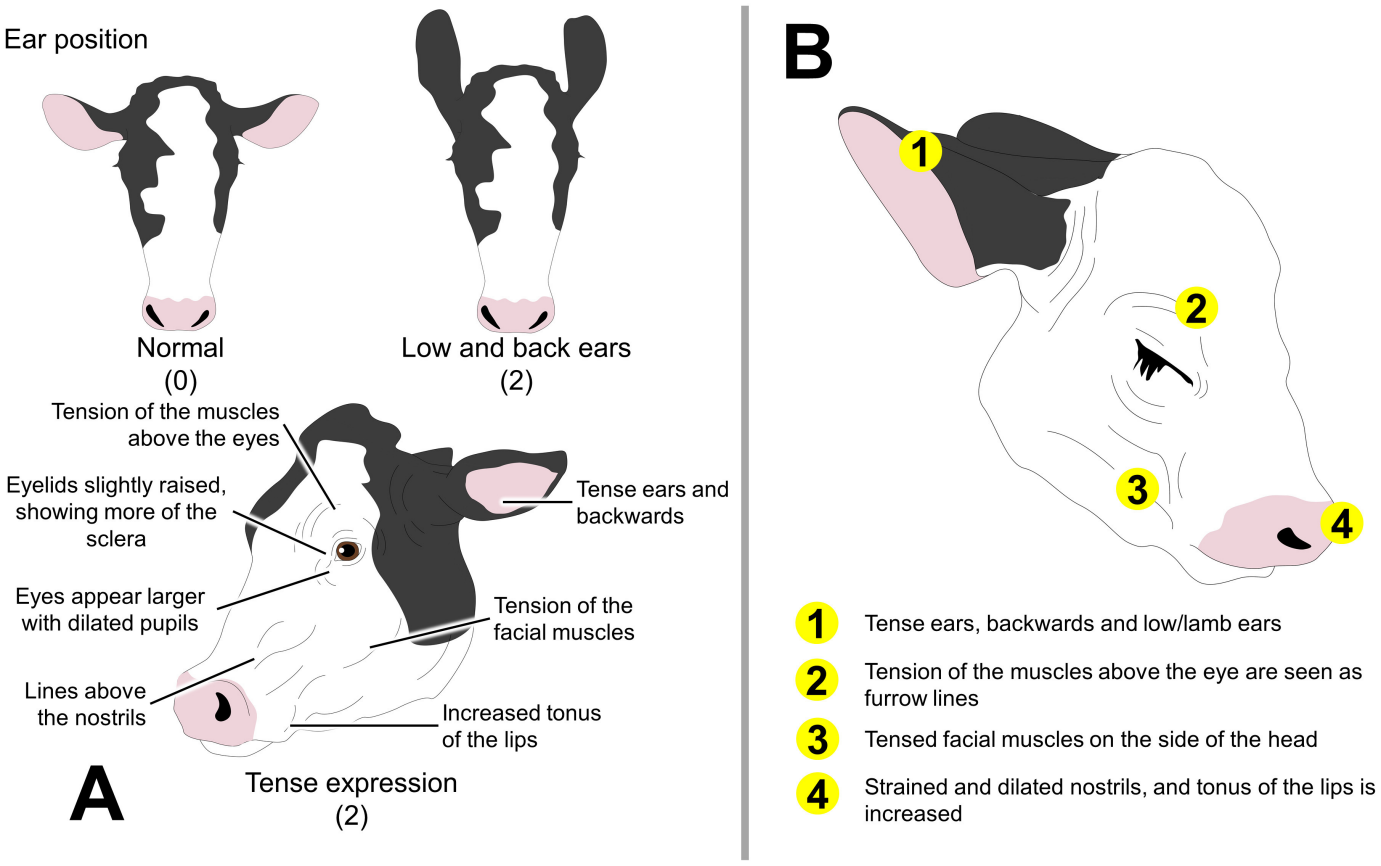
Facial expression related to pain in cattle and calves. **(A)** The Cow Pain Scale. Although this behavioral-based assessment method does not focus on facial expression, a tense facial expression and changes in ear position are included in the evaluation of pain. Numbers inside the parenthesis represent the pain score. **(B)** Calf Grimace Scale and the main FAU.

Although several studies have established scales to detect pain in cattle using behavior (e.g., the Unesp-Botucatu Cattle Acute Pain Scale) (86), limited studies have focused on the facial changes present in cattle or calves with pain. For example, the Cow Pain Scale considers several behavioral aspects and body language traits but also includes ear position and facial expression (neutral look or tense expression) to rate pain on a scale of 0 to 2 (57) without being exclusively focused on facial changes (Figure 3).

Some other studies have reported changes in facial expression in castrated bulls, including modifications of the eye region such as tension and convex appearance of the orbicular muscle of the eye (*orbicularis oculi muscle*) (87). In this study, each FAU was scored with an intensity of 1 to 5 to compare surgical, chemical, and immunological castration techniques. The highest scores were reported in animals undergoing surgical castrations (3–5 points) in contrast to chemical (1–5 points), and immunological techniques (0–3 points) (87). In Nellore and crossbred beef cattle, Müller et al. (88) compared the facial expression of animals before and during branding with hot iron. The findings included a higher frequency of mouth opening, backward ears, dilated nostrils, raised inner brow, and raised outer brow in animals experiencing pain. In the same procedure but in calves, Hernandez et al. (89) used changes in facial expression such as eye white showing, tension in the upper eyelid muscle, contraction of the orbicular muscle of the eye, third eyelid protrusion, tension in the masticatory muscles, tension of the muzzle, and mouth opening to compare the effect of analgesia administration in hot iron branded calves. The results showed that only “tension of masticatory muscles” was significantly different between animals with and without analgesic treatment (up to 1.57 ± 0.71 during branding), suggesting that this parameter is a more sensitive indicator of pain during branding.

These studies show that, although no grimace scale has been adapted to bulls and cattle, significant facial changes are observed in bovines, which might help to develop and validate further scales for use in several painful conditions. An example is Ginger et al. (90) study, in which mastitis pain was evaluated through 29 FAUs referring to the orbital, auricular, and mouth and muzzle regions. After the induction of mastitis, the cows displayed nostril dilation and decreased the motion of the muzzle and the opening of the eyes.

Recognizing that these FAUs change in animals experiencing pain could help identify facial changes for assessing farm procedures, considering the role of upper (levator muscle palpebrae superior, levator muscle of the medial angle of the eye) and lower eyelid muscles (levator muscle of the lateral eye angle, malar muscle) to open the eyes.

In the case of calves, routine procedures such as disbudding, castration, and tail docking are often performed without proper pain management (91–93). Thus, Farghal et al. (94) developed the Calf Grimace Scale (CGS) in Angus animals, where each FAU has a maximum score of 2 (Figure 3). The authors assigned six FAUs such as (1) ear position; (2) orbital region opening; (3) tension above the eye; (4) nostril dilation; (5) straining of chewing muscle; and (6) mouth opening to assess pain after castration. Higher CGS scores were registered after castration (from 0.25 to 0.50). Moreover, it was found that adverse external factors such as changes in the environment, dam separation, and restraint increase the presentation of FAUs related to pain, which needs to be considered when adopting the CGS in other settings.

Although some grimace scales might have limitations and have not yet been developed in all species, the aforementioned studies show that specific facial changes occur in animals experiencing pain. These alterations can be utilized to assess pain intensity and even aid in the pharmacological management of pain.

## 4 Grimace scales and facial expression related to pain in laboratory animals

Many studies that use animal models may involve pain, or discomfort to some extent as a direct consequence of invasive approaches. Thus, due to the ethical and legal requirements for using laboratory animals, the first grimace scales were developed to study pain in laboratory animal models (95). In addition to its ethical relevance, understanding pain is important because it impacts the reliability and translatability of results in experimental protocols (96). To date, grimace scales in laboratory animals can evaluate both acute and chronic conditions. Acute pain includes post-surgical pain, dental procedures, and traditional pain models (16). For chronic conditions, scales have been used to evaluate osteoarticular pain, models of neuropathic pain (e.g., migraine, spinal cord injury, stroke), cancer, and visceral pain (96).

### 4.1 Mouse Grimace Scale

**Figure 4:**
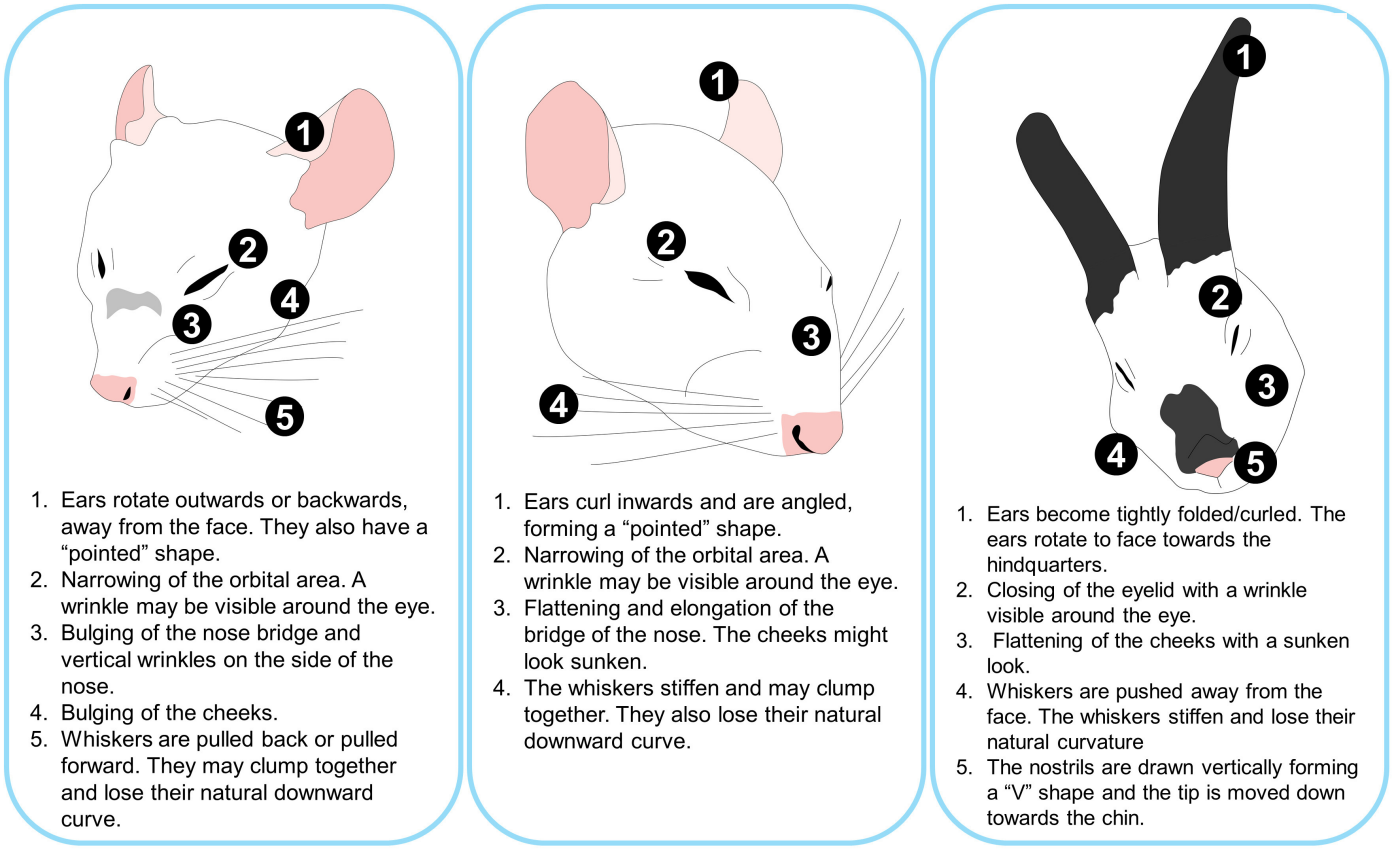
Main changes observed in the facial expression of mice, rats, and rabbits when perceiving severe pain.

Langford et al. (24) developed and validated the Mouse Grimace Scale (MGS). Five FAU are used to identify pain in different settings in mice: (1) orbital region opening; (2) nose bulge (a nasal protuberance appearance); (3) ear position; (4) ear position; and (5) changes observed in the position of the whiskers. Using a scale from 0 to 2, where 0 is “not present,” 1 is “moderate,” and 2 is “obvious” pain, each FAU is rated to obtain an overall grimace score (24). Figure 4 summarizes the facial expression of pain in mice and other laboratory animals.

Most studies using the MGS focus on modeling human pain-related conditions, as well as assessing pain during husbandry or veterinary procedures, and exploring biological differences between animals (96). An example is the study of Matsumiya et al. (97) which used the MGS to evaluate pain during the postoperative period after laparotomy. The authors found that postsurgical pain can last up to 48 h based on grimacing. They also reported that buprenorphine was an effective analgesic in reducing pain signs, whereas acetaminophen was ineffective. Similar to these findings, during invasive procedures such as craniotomy, Cho et al. (98) used the MGS to assess the analgesic efficacy of carprofen, meloxicam, and buprenorphine and if the route of administration (whether injectable or oral) influenced pain relief. According to a lower maximum score during the first 72 h after surgery (0.3), injected analgesics were more efficient in alleviating postsurgical pain than oral administration (0.4). Moreover, regardless of the route, buprenorphine had the lowest pain scores (0.2) in contrast to saline (0.4) (98). This is relevant because current practices recommend the use of analgesics through the drinking supply. However, according to the MGS, it might not provide a correct pain treatment. Additionally, in other procedures such as vasectomy, scores for the MGS did not differ between mice treated with meloxicam and bupivacaine, showing that both treatments are adequate to treat acute postsurgical pain (99).

These studies show that MGS does not only help to recognize the presence/absence of pain but can also discriminate between treatments, serving as a non-invasive method to assess the efficacy of the pain management protocol. This is helpful, particularly when the MGS is used together with other methods to evaluate pain, such as behavior, where the MGS has shown a strong positive correlation with pain-related behaviors including arching, circling, falling, flinching, staggering, twitching, and writhing (*r* = 0.93), meaning that these behaviors were frequently observed in animals with high scores of pain (99).

As Miller and Leach (100) mention, the MGS has limitations. Differences in pain scores have been reported based on the strain and sex of the mice. Even during baseline evaluations, males tend to score higher than females. However, other authors argue that the MGS is reliable for assessing anesthesia with ketamine/xylazine, reporting good agreement (ICC = 0.85), particularly for orbital region opening, whereas the nasal protuberance and cheek tightening showed the lowest agreement. Moreover, it was also reported that evaluators with experience gave higher scores to animals, which needs to be considered for further research and application of the MGS in a clinical setting (101). Similarly, when the MGS was used to evaluate analgesic protocols during ear notching, although it is known as a painful husbandry procedure, the grimace scale was not able to detect any facial changes (102). These results emphasizing the importance of using other methods to identify pain and potentially assess the effectiveness of analgesics in laboratory mice.

Codifying pain through the MGS can be time-consuming and labor-intensive for researchers. Thus, current machine learning techniques combine automatic methods of face detection and grimace detection in laboratory animals. For example, Vidal et al. (103) analyzed the eye region using the grimace scale as a basis, showing promising results in automatically detecting pain faces in furred mice. Ernst et al. (104) automatized pain detection in mice undergoing intraperitoneal injection of carbon tetrachloride. From 609 pictures analyzed, the authors found that machine learning can help to select appropriate images to be rated based on the quality (e.g., with all regions of the FAU visible and focused pictures). Additionally, an accuracy of up to 99% has been observed when using automatic face detection to identify mice perceiving pain after anesthesia and surgery (105). Thus, combining the MGS with other pain evaluation methods and automatization of the technique is a promising tool for pain assessment in mice.

### 4.2 Rat Grimace Scale

Contrarily to the MGS, the Rat Grimace Scale (RGS), developed by Sotocinal et al. (23), has only four FAU: (1) orbital region opening; (2) Nose/cheek flattening; (3) ear changes; and (4) whisker changes. This was adjusted after detecting that nose and cheek flattening always occur at the same time in rats with pain, an event that is not present in mice. Using a similar score of 0 to 2 (from absent to severe pain), this scale rates the intensity of the facial expression (106). Further studies established an analgesic intervention threshold where RGS scores above 0.67 represent animals that require rescue analgesia (107).

Using the RGS, researchers have identified pain during various surgical procedures such as spinal surgeries, and models of visceral, orthopedic, and inflammatory pain (108). An example of this is the application of the RGS to discriminate between animals receiving analgesia (buprenorphine, combination of buprenorphine and meloxicam) and saline administration followed by intra-plantar administration of carrageenan (108, 109). The authors concluded that the RGS obtained acceptable limits of agreement as a feasible method to assess pain. Similarly, it has been used to evaluate analgesic protocols for neuropathic pain in rodent models of cervical radiculopathy. When comparing the RGS score of the sham group with the animals undergoing cervical nerve root compression, the injury by itself significantly increased the scores (sham: 0.93 ± 0.20; injury: 1.27 ± 0.18). Moreover, administration of meloxicam decreased the RGS score to sham levels at 6 h after the procedure (109).

Apart from acute pain, some studies have tried to implement both the MGS and RGS to chronic painful conditions such as trigeminal neuropathic pain models. In this sense, Akintola et al. (110) used a murine model (with mice and rats) of constriction injury of the infraorbital nerve to imitate neuropathic pain and obtained pain scores using facial expression. The authors found that both grimace scales had higher scores in animals undergoing constriction injury (average of 1) and these results were related to lower withdrawal thresholds from mechanical stimuli, suggesting that grimace scales are sensitive to assess chronic pain. The RGS has also been compared or used together with behavioral-based scales, as reported by Klune et al. (111), who compared the RGS with the composite behavior score after laparotomy. The results showed that the scores of both scales significantly increased after the surgical procedure in animals where analgesia was not provided (saline); however, the RGS registered that pain scores decreased only with the administration of buprenorphine, showing that both methods have different sensitivity.

Similar to the MGS, automated methods have been tested with the RGS, recording a precision and accuracy of 97 and 93%, respectively (112). Furthermore, during other events that might cause pain, such as euthanasia, Domínguez-Oliva et al. (113) implemented the RGS to assess the quality of euthanasia. It was found that inhalational agents (e.g., CO_2_ and isoflurane) recorded the highest scores (up to 1.20). However, other authors have focused on the limitations of the RGS, such as the use of inhalant anesthetics, including isoflurane. In this sense, Miller et al. (114) found that isoflurane increases the RGS score (~up to 2.5) and this needs to be considered when evaluating pain in trials using these drugs. Although the RGS can have limitations (as any other grimace scale), assessing pain in rats is a non-invasive alternative to refine the management of laboratory animals.

### 4.3 Grimace scales for rabbits and ferrets

Apart from mice and rats, other grimace scales have been developed for species that are frequently integrated in biomedical research. Keating et al. (25) developed the Rabbit Grimace Scale (RbtGS) to evaluate the pain elicited during ear tattooing, a routine procedure. The RbtGS uses orbital region opening, cheek flattening, nostril shape, whisker shape and position, and ear shape and position as the five FAUs to rate pain. An animal with obviously present signs of pain has closed eyes, flattening of the cheeks, nostril shaped vertically as a “V,” stiff whiskers, and ears rotated toward the pelvic limbs (hindquarters). This scale has been used to recognize pain after surgical castration by Miller et al. (115). In this study, RbtGS recognized that scores of up to 4.5 within the first 5 h after orchidectomy were related to pain, an effect that was lessened with the use of meloxicam in combination with lidocaine (score of 3). To improve the application of the RbtGS, a composite pain scale for rabbits (CANCRS) has been developed to rate pain as not present, discomfort, moderate, and severe pain (116). However, as Shaw et al. (117) highlight, to use grimace scales, the evaluator must know the species and the facial anatomy of animals to accurately assess pain and standardize the use of the RbtGS and other facial scores.

Other grimace scales developed but that have not been extensively used are the Ferret Grimace Scale (FGS) and the Guinea pig Grimace Scale (GGS). With five FAUs, the Feret Grimace Scale (FGS) was developed by Reijgwart et al. (118), testing its ability to recognize pain during surgical implantation of telemetry. The authors reported that orbital region opening was the most accurate FAU to rate pain in the species; however, further studies are required to evaluate the application of the FGS during surgical procedures or other painful events.

In laboratory animals, pain evaluation using facial expression is considered a refinement method to improve animal's welfare and is a valuable tool for its real-time application and rapid identification of pain.

## 5 Grimace scales and facial expression related to pain in companion animals

### 5.1 Grimace scales in cats

**Figure 5:**
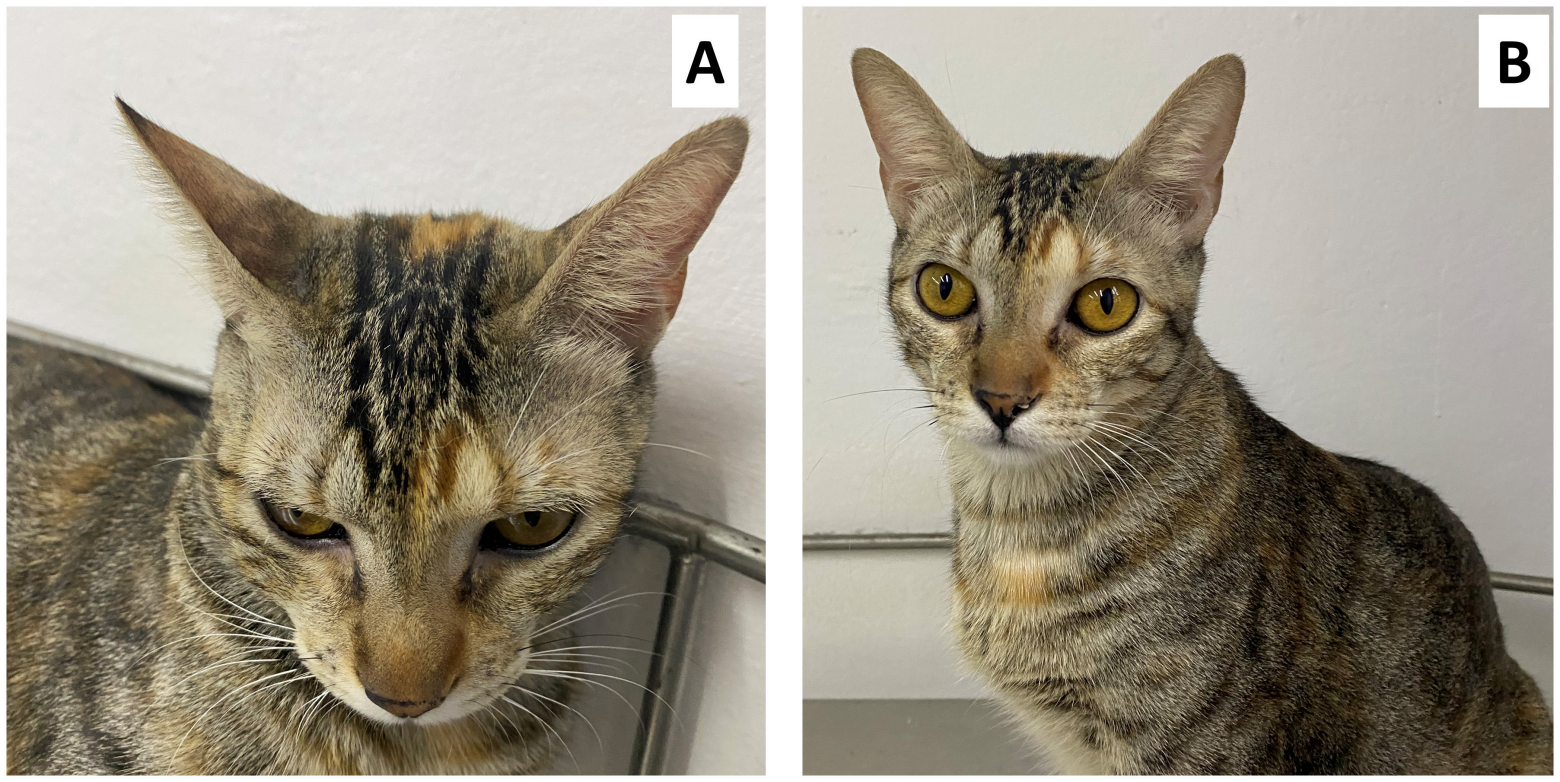
Four-year-old female domestic cat diagnosed with acute pancreatitis. **(A)** shows the flattening of the ears and the position of the whiskers forward, as well as the squinting of the eyelids, these being grimaces suggestive of moderate pain according to the FGS. In **(B)** you can see the same patient without pain after the administration of analgesics.

As companion animals are the closest species to humans, the study of pain in these species has also focused on non-invasive alternatives to recognize pain, such as facial expressions, particularly in cats. Cats are one of the species with the greatest advances in the study of facial expressions to identify their emotional state and to maintain animal welfare (119, 120). Evangelista et al. (26) designed and validated the Feline Grimace Scale (FGS), identifying five action units: (1) flattened ears; (2) tension of the orbital muscles; (3) tension of the lips; (4) position of whiskers; and (5) head position, with a maximum score of 1 (Figure 5). Using the FGS, the feline pain expression was characterized as a cat with flattened ears, squinted eyes, tense muzzle, straight and forward whiskers, and a head below the shoulders. The same authors also used the FGS to evaluate the response to an analgesic treatment, finding that the scores decreased from 0.72 to 0.44 (26).

Further studies have applied the FGS to test its clinical applicability in real-time during surgical procedures to monitor the requirements of rescue analgesia. In this sense, cats were evaluated during the postsurgical period after undergoing ovariohysterectomy, where scores of 0.47 ± 0.24 and 0.45 ± 0.19 decreased to 0.21 ± 0.18 and 0.18 ± 0.17 after the administration of analgesia (121). Moreover, Cheng et al. (122) determined that the FGS can be used in kittens undergoing ovariohysterectomy with or without multimodal analgesia. The authors reported that the FGS total ratio score increased at 1 and 2 h after the surgery (IQR of 0.30) from baseline IQR of 0.10 and that analgesia administrations decreased the FGS total ratio (IQR from 0.40 to 0.20). Although these studies suggest the application of the FGS after surgical interventions, Watanabe et al. (123) mention that sedation increases the FGS scores. This was shown in healthy cats anesthetized with dexmedetomidine-butorphanol and propofol-isoflurane protocols, in whom increases up to 0.51 ± 0.05 and 0.34 ± 0.05, respectively, were observed. Thus, although the FGS can evaluate pain, the physiology of the motor control of facial muscles must be considered.

In other experimental trials, the reliability of the FGS when used by the animal's owner or caregivers has been also evaluated. In this sense, Evangelista et al. (56) evaluated the agreement between owners, veterinarians, technicians, and nurses when using the FGS. The results showed that all groups were able to reliably score changes in the five FAUs, but the muzzle and whiskers yielded low reliability (0.39–0.74). Similarly, Monteiro et al. (124) reported that owners assigned similar overall FGS scores than veterinarians (caregivers: 0.9 ± 0.0; veterinarians: 0.7 ± 0.1) with good reliability for the ears, eyes, and head (ICC of 0.65, 0.69, and 0.65, respectively). These studies are relevant because, generally, grimace scales are used by veterinarians and training is recommended to accurately assess pain. However, in companion animals, the owners are the main individuals who can recognize changes in behavior and body language—including facial expressions—before consulting a professional.

Another scale that does not focus on changes in the facial expression of cats but includes an indicator related to facial changes is the Unesp-Botucatu Multidimensional Feline Pain Evaluation Scale (UFEPS–SF). This scale mostly considers changes in the posture and behavior of cats after surgery; however, the FAU “eyes partially closed” is included in the scale to evaluate pain with a maximum score of 12 (125, 126). Therefore, in the case of domestic cats, the FGS represents a highly useful tool to determine pain and monitor its management not only in a clinical setting but for the owners as well.

### 5.2 Grimace scales in dogs

In dogs, unlike cats, the study of facial expression and emotional states—including pain—is still ongoing. Facial expressions in dogs have been investigated to suggest certain emotions such as happiness, sadness, fear, anger, disgust, and surprise in certain social conditions (38, 127). However, their meaning and presentation are highly influenced by domestication, causing anatomical differences according to the breed that modify the entire interpretation of the facial expression. Moreover, traits such as the shape and position of the ears and the color of the coat play an important role in accurately identifying facial movements and their intensity (51).

Although there is no developed and validated grimace scale for dogs, similar to what was observed in other species, current behavioral-based pain scales include some aspects related to facial expression. An example is the Colorado State University Canine Acute Pain Scale (CSU-CAPS), which considers facial changes such as droopy ears, arched eyebrows, and darting eyes (known as a “worried facial expression”) to assess the level of pain from 0 to 4 (minimal to severe pain), considering a value >2 as a rescue point (128). The CSU-CAPS does not focus on facial expression but has been extensively used to evaluate pain in patients with dermatologic, neurologic, and orthopedic disorders (129).

## 6 Reliability of grimace scales

Grimace scales rely on the observer's ability to identify the intensity of the facial changes when animals perceive pain. Therefore, a critical aspect to consider when using these scales is the method's reliability, which is referred to as repeatability and consistency even when evaluated by different observers (64). Several papers on different species have addressed this issue, as shown in horses. In horses with colic pain, Rosenzweig et al. (130) reported excellent interobserver reliability (ICC = 0.86), with a sensitivity and specificity of 100 and 79%, respectively. However, when using the HGS to evaluate dental pain in horses, Sidwell et al. (131) reported poor interobserver reliability (ICC = 0.27) for assessing chronic pain. Similarly, another study evaluated animal welfare indicators in horses such as the HGS and behavioral traits, finding acceptable to good reliability [bias-adjusted kappa (PABAK) from 0.4 to ≥ 6] except for the tension above the eye area and orbital region opening (132). Regarding this, the authors discussed that differentiating between the absent and moderate presence of FAUs might be challenging for non-trained observers, which suggests that including a more detailed description of the score might improve the assessment through the HGS. This was also discussed by Dai et al. (64), who tested if a 30-min training program on HGS improves assessors' agreement. In this study, the training was provided to undergraduate students with no horse experience. The scores of these students before and after the training were compared to those of an HGS expert. According to Cohen's k coefficient, pre-training agreement ranged from 0.20 to 0.68 for tension above the eye area and stiffly backwards ears, respectively. Post-training agreement increased to 0.90 and 0.91, respectively for each FAU; however, a high variability of agreement was found for the other FAUs, suggesting that 30 min might not be sufficient to completely improve the performance of naïve observers and additional sessions might be required.

In the case of rodents, similar to horses, Hohlbaum et al. (101) reported that some individual FAUs record better agreement levels than others. Using the MGS before and after anesthesia in mice, the authors found an overall good interrater agreement (ICC = 0.85); however, the best (ICC = 0.88) and poorest agreements (ICC = 0.15) were found for orbital region opening and nose and cheek bulging, respectively. An excellent ICC (0.90) was also reported by the developers of the MGS, with a global accuracy of up to 81% when rating mice after the pain assay with the acetic acid abdominal constriction test (24). For the RGS, a study reported the effect of re-scoring images from rats under acute pain models such as intraplantar carrageenan, plantar incision, and Complete Freund's adjuvant. This study found that no trainee raters increased the ICC after re-scoring from moderate (0.58) to very good (0.85). Moreover, the ability was retained 4 years later, obtaining a very good inter-rater and intra-rater reliability (0.82 and 0.87, respectively). These examples show that the grimace scales are reliable in assessing different types of acute pain, particularly if training is involved. However, Whittaker et al. (96) emphasize that the good-to-excellent interrater agreement might be related to the fact that only 50% of the published reports using MGS include reliability assessment or do not perform this analysis. This is critical because, in a clinical scenario, a single observer performing the scoring is not always feasible and might reduce the applicability of the MGS.

The importance of assessing the effect of different evaluators has also been reported in cats, where the knowledge of the owners, veterinarians, veterinary students, and nurses might differ. Regarding this, Evangelista et al. (56) found good inter-rater reliability above 0.8, an excellent intra-rater reliability for students and veterinarians (ICC = 0.91), and a very good agreement between all groups and veterinarians when using the FGS. Similarly, the FGS was able to detect naturally occurring acute abdominal pain with good inter-rater reliability (0.89) and excellent intra-rater reliability (ICC = 0.91) (26). Watanabe et al. (133) also registered the inter-rater reliability in cats undergoing dental extractions. The authors found good inter-rater reliability (0.84), particularly for ears (0.68) and orbital region opening (0.76). Additionally, no difference or influence was found in FGS scores when the owner was present, a finding that was different from what Adami et al. (134) reported in cats undergoing neutering. In these animals, only 59% of paired observations had a fair level of agreement between assessors, and 44% of the observations had good reliability. These studies suggest that tools such as the FGS are useful for rating animal pain, the main limitation of the grimace scales is the subjectivity that might be related to the expertise level of the evaluators. Moreover, as mentioned in other species, certain FAUs show greater intra-rater reliability in the FGS, such as the eyes and ears, while muzzle and whiskers had moderate reliability (56). This means that it might be harder for non-trained (e.g., owners) and trained (e.g., veterinarians) observers to identify and accurately detect changes in these FAUs.

As reported by Evangelista et al. (60), currently, the MGS, RGS, HGS, and FGS are the scales with the highest level of evidence, while the SGS and the EQUUS-FAP had the lowest. The level of reliability and responsiveness of the scales depends on factors such as the observer's experience with the species to recognize the appropriate facial features and changes (101). Moreover, most of the studies referring to grimace scales show results based on images. The real-time applicability of grimace scales and their inter-observer reliability need to be considered in future studies. Therefore, further studies must consider methods to improve the reliability of the scales, such as incorporating precision livestock techniques, automated methods, or artificial intelligence.

## 7 Perspectives

Scientific evidence suggests that facial expression is a valuable tool to recognize animal pain. However, there are still areas of research that need to be considered. For example, the study of facial expression requires characterizing the FAUs that are related to pain from those that might reflect other emotional states such as anxiety or fear (26, 58, 135–137). This has been mentioned by Werner et al. (138) in horses, who proposed anatomically described FAUs to improve the reliability of the HGS (e.g., replacing the term “ear” with “pinna” and referring to the orbital region).

When using facial expression scales, although most species share the same FAU, it is important to recognize the differences in facial expressions according to the species. For example, all species with a validated grimace scale show closing of the orbital region when in pain –particularly when the pain is severe or when the change is obviously present–. However, in some species, it is harder to recognize these changes (e.g., piglets and pigs). Although pigs' eyeball size is the same as humans (139), the eyes appear to be smaller and might be partially covered by their hanging ears, which can make it difficult to recognize the closure of the eye (21, 140). In rodents, “nose bulge” refers to a protuberance that appears in the dorsum of the nose (23, 100). In contrast, “nose bulge” in piglets and pigs refers to several skin folds above the snout (141). In the same species, the facial pain scale developed for sows has significant differences with the PGS regarding the tension above the eyes. In piglets, eyes completely or partially closed and fully tightened are considered indicative of pain (141). In contrast, in sows, eyes completely open with the ocular sclera visible is an indication of severe pain (2 points) (78). Thus, within species, the age and the painful condition must be considered.

Additionally, one of the main practical implications of facial pain scales is the opportunity to use them in clinical or on-farm situations. Although the evaluator needs training to properly identify changes in the facial expression of animals (142, 143), several scales can be used by veterinarians, stock people, and owners to identify pain. In companion animals, studies have shown that cat owners can reliably identify facial changes in the position of the ears and orbital region opening in animals with painful conditions including pancreatitis, cystitis, ovariohysterectomy, etc. (133). As previously mentioned, the pain scale developed for sows can be used during farrowing to potentially identify eutocic and dystocic processes by trained observers (78). Moreover, facial pain scales for bovines, equines, and ovines can be used during castration, spontaneous colic, mastitis, and footrot cases (22, 62, 87), representing a non-invasive alternative for farm personnel to promptly detect pain in domestic animals.

The development of additional grimace scales is another field where further studies are required. For example, although dogs are the main companion animal for humans, to date, there is not a grimace scale specifically designed for the species. In dogs, characteristics such as coat length, color, or facial traits might influence the evaluation. In the case of guinea pigs (frequently used as animal models in research), preliminary data suggest that facial expression might not be able to be evaluated in the species. Moreover, current grimace scales are designed for adult or juvenile animals. Future studies should consider if neonates have the same changes and FAUs.

## 8 Conclusions

Grimace scales in animals are valuable tools for assessing facial expressions associated with pain; however, their interpretation can be challenging due to the multifactorial nature of facial changes. The grimace scales developed in several species can accurately evaluate the degree of pain in animals exposed to different noxious stimuli via changes in FAUs such as eye narrowing, muzzle tension, and ear position. However, although indicative of nociception, some FAUs may also reflect anxiety or other negative emotional states such as stress. The association between the nociceptive pathway and the efferent motor responses is the anatomo-physiological basis of facial expression related to pain in mammals. As the anatomy of the face differs according to the species, species-specific FAUs need to be considered in each case. Moreover, the factors that can alter facial expression and pain evaluation must also be considered, such as coat characteristics or anesthetic drugs. Although grimace scales are becoming more popular for both clinicians and tutors as a non-invasive method to identify pain, it is important to mention that knowledge about the “normal facial expression” of a species is required to accurately assign scores in animals to evaluate acute pain and provide wellbeing in domestic animals.
